# Non-Apoptotic Programmed Cell Death: From Ultrastructural Characterization to Emerging Therapeutic Opportunities

**DOI:** 10.3390/cells15020111

**Published:** 2026-01-08

**Authors:** Philip Steiner, Lena Wiesbauer, Hubert H. Kerschbaum, Susanna Zierler

**Affiliations:** 1 Institute of Pharmacology, Faculty of Medicine, Johannes Kepler University Linz, 4020 Linz, Austria; lena.wiesbauer@jku.at (L.W.); susanna.zierler@jku.at (S.Z.); 2 Department of Biosciences and Medical Biology, Paris Lodron University Salzburg, 5020 Salzburg, Austria; hubert.kerschbaum@plus.ac.at; 3 Walther Straub Institute of Pharmacology and Toxicology, Ludwig-Maximilians-Universität München, 80336 Munich, Germany; 4 Clinical Research Institute for Inflammation Medicine, Johannes Kepler University Linz, 4020 Linz, Austria

**Keywords:** programmed cell death, non-apoptotic cell death, autosis, ferroptosis, caspase-independent cell death, ultrastructure, autophagy, regulated cell death, cancer, neurodegenerative diseases

## Abstract

**Highlights:**

**What are the main findings?**

- Non-apoptotic programmed cell death comprises diverse modalities such as ferroptosis, necroptosis, pyroptosis, autophagy, paraptosis, autosis, and more, with each modality characterized by unique ultrastructural hallmarks and molecular signaling pathways that distinguish them from classical apoptosis.
- Comprehensive morphological characterization reveals distinct intracellular features for each non-apoptotic PCD modality, ranging from mitochondrial cristae reduction in ferroptosis to perinuclear space ballooning in autosis, facilitating their identification via advanced microscopy techniques.
- Non-apoptotic PCD pathways offer dual therapeutic potential: induction strategies can overcome apoptosis resistance in cancer therapy, while inhibition approaches provide cytoprotection in neurodegenerative diseases and myocardial injury.

**What are the implications of the main findings?**

- Targeting non-apoptotic PCD pathways opens novel therapeutic avenues for apoptosis-resistant malignancies through pathway-specific nanomedicines and pharmacological modulators that selectively eliminate cancer cells while sparing normal tissue.
- Understanding ultrastructural hallmarks of non-apoptotic PCD enables precise mechanistic differentiation in disease models, supporting development of pathway-specific biomarkers and precision medicine approaches for cancer, neurodegeneration, and inflammatory diseases.
- The comprehensive characterization of non-apoptotic PCD modalities provides important templates to identify and manipulate these signaling pathways, potentially enabling the establishment of treatment strategies for a wide range of pathological conditions, from tumor immunity to tissue homeostasis.

**Abstract:**

Distinct forms of non-apoptotic programmed cell death (PCD) play a central role in human and animal health and their signaling cascades provide pharmacological targets for therapeutic interventions. Non-apoptotic modalities of programmed cell death include well characterized forms, such as ferroptosis, necroptosis, pyroptosis, autophagy, paraptosis, as well as newly characterized varieties, such as cuproptosis, disulfidptosis, and erebosis. Each pathway exhibits unique molecular signaling signatures, ultrastructural characteristics, and functional outcomes that distinguish them from classical apoptosis. While pharmacological targets in the signaling cascade are promising objectives for overcoming apoptosis resistance in cancer therapy, inhibition of cell death in the myocardium or nervous system is critical for cytoprotection. This review provides detailed characterization and schematic visualization of cellular and subcellular hallmarks for each non-apoptotic PCD modality, facilitating their morphological identification. Understanding these diverse pathways is crucial for developing innovative therapeutic interventions in cancer, neurodegeneration, and inflammatory diseases.

## 1. Introduction

The understanding of programmed cell death (PCD) has changed remarkably in recent years. It has evolved from the traditional apoptosis-centered paradigm to a diverse spectrum of non-apoptotic PCD pathways that are reshaping biomedical research [1,2]. The Nomenclature Committee on Cell Death (NCCD) formally recognized twelve distinct non-apoptotic regulated cell death subtypes in 2018, highlighting the mechanistic complexity beyond classical apoptosis [1]. Among these, ferroptosis, an iron-dependent form of cell death characterized by lipid peroxidation and glutathione peroxidase 4 (GPX4) inactivation, has emerged as a pivotal therapeutic target since its discovery in 2012 [3,4,5]. Pyroptosis, mediated by gasdermin family proteins and inflammasome activation, represents another crucial inflammatory cell death pathway that forms membrane pores and releases proinflammatory cytokines [6,7,8]. Necroptosis, orchestrated by the RIPK1/RIPK3/MLKL signaling cascade, provides an alternative PCD mechanism when apoptotic pathways are compromised [9,10]. More recently identified pathways include cuproptosis (copper-induced mitochondrial dysfunction) and parthanatos (PARP1-dependent cell death), each offering unique therapeutic opportunities [2,11]. The conceptualization of PANoptosis, the coordinated activation of pyroptosis, apoptosis, and necroptosis, has further illuminated the interconnected nature of these pathways [7,12]. Such non-apoptotic modalities have shown important clinical significance, particularly in overcoming or circumventing apoptosis resistance in cancer therapy, enhancing immunogenic cell death responses, and treating neurodegenerative diseases where oxidative stress predominates [2,13,14]. This dual therapeutic potential highlights the contrasting pharmacological objectives in PCD research: while cancer treatment aims to promote cell death to eliminate malignant cells and overcome apoptosis resistance, neurodegeneration therapy focuses on cytoprotective strategies to preserve vulnerable neurons and prevent excessive cell loss. The convergence of nanotechnology-based drug delivery systems or nanomedicines, with non-apoptotic PCD mechanisms, has opened up numerous possibilities for targeted therapeutic interventions. The development of pathway-specific nanomedicines, such as ferroptosis-inducing iron oxide nanoparticles or necroptosis-activating RIPK1-directed nanocarriers, illustrates the translational potential of this expanding field [15,16,17]. As the increasing importance of non-apoptotic PCD mechanisms is constantly evolving, with new modalities being described and PCD “affiliations” continuously shifting, a current and comprehensive overview of this topic is invaluable. The aim of this review is to provide such an overview and, in addition, to characterize and schematically visualize the known cellular and subcellular hallmarks of the various non-apoptotic PCDs.

## 2. The Importance of Non-Apoptotic Programmed Cell Death

Non-apoptotic PCD has emerged as a crucial area of research in developmental biology as well as neurodegenerative and cancer therapeutics. Many cancer cells develop resistance to apoptosis, limiting the effectiveness of traditional cancer therapies. Non-apoptotic forms of PCD provide alternative pathways to eliminate cancer cells that have become resistant to apoptosis, offering new opportunities for developing cancer therapies, particularly for tumors resistant to apoptosis-inducing treatments [13,18]. Non-apoptotic PCD and the mechanisms behind it play crucial roles in animal development, particularly in the formation of the nervous system, germline, and gonadal structures. Their importance is further emphasized by the fact that they appear to be under strong evolutionary pressure [19]. Non-apoptotic PCD pathways can serve as backup programs when caspases (key enzymes in apoptosis) are inactivated or unavailable. Unlike apoptosis, which often leads to immune tolerance, some forms of non-apoptotic PCD, for example, necroptosis and pyroptosis, can trigger potent immune responses [13]. In particular, ferroptosis, autophagy, and pyroptosis are involved in different molecular mechanisms and cellular processes, allowing for a more differentiated regulation of cell death in different contexts [18,20]. To better understand these diverse signaling pathways and their role in the development and progression of diseases, a more nuanced and comprehensive understanding of the underlying cell biology is needed. This, in turn, can serve the potential development of targeted therapeutic approaches. A crucial step in this direction is a better differentiation from apoptosis.

## 3. Comparison Between Apoptotic and Non-Apoptotic Programmed Cell Death

The comparison between non-apoptotic PCD and apoptosis is critical for understanding their distinct roles in pathophysiology and therapeutic targeting. Non-apoptotic PCD mechanisms exhibit unique molecular signatures and functional outcomes compared to classical apoptosis [21,22,23]. While apoptosis relies on caspase activation, chromatin condensation, and apoptotic body formation, necroptosis, for example, is mediated by receptor-interacting protein kinase (RIPK) activation and plasma membrane rupture independent of caspases, with phosphorylated mixed lineage kinase domain-like protein (MLKL) serving as a key biomarker. Pyroptosis, characterized by gasdermin-mediated pore formation and interleukin release, links cell death to inflammatory responses through inflammasome activation, contrasting with apoptosis’ immunologically silent clearance [4]. Ferroptosis diverges further through iron-dependent lipid peroxidation driven by Fenton reactions, causing mitochondrial membrane disruption without nuclear fragmentation [24]. These pathways exhibit divergent immunological consequences: apoptosis promotes tolerance, whereas non-apoptotic PCD such as necroptosis and pyroptosis trigger inflammation or tissue repair signals, influencing outcomes in cancer and neurodegeneration [2,21,22]. Therapeutically, targeting ferroptosis has shown efficacy in eliminating apoptosis-resistant tumors by exploiting iron metabolism dysregulation in epithelial–mesenchymal transition (EMT)-positive cancers [2,24]. Crosstalk between pathways, such as caspase-8-mediated suppression of necroptosis via RIPK1 cleavage, highlights regulatory complexity and hierarchical control in PCD execution [21,22]. Advances in single-cell omics and biomarkers like lipid peroxidation products (e.g., malondialdehyde) now enable precise mechanistic dissection in disease models, revealing context-dependent roles in tumor immunity and neurodegeneration [2,4,24]. This rapidly evolving topic highlights the need for pathway-specific modulation to unlock the therapeutic potential of non-apoptotic PCD subtypes while mitigating off-target inflammatory effects. For instance, necroptosis-inducing agents like shikonin show promise in non-small-cell lung cancer, while nanoparticle-based ferroptosis inducers selectively target iron-rich malignancies [13]. Such strategies underline the importance of cellular and molecular differentiation of PCD mechanisms for optimizing precision medicine approaches in biomedical research [2].

## 4. Characterization and Pharmacological Modulation of Non-Apoptotic Programmed Cell Death

**Figure 1:**
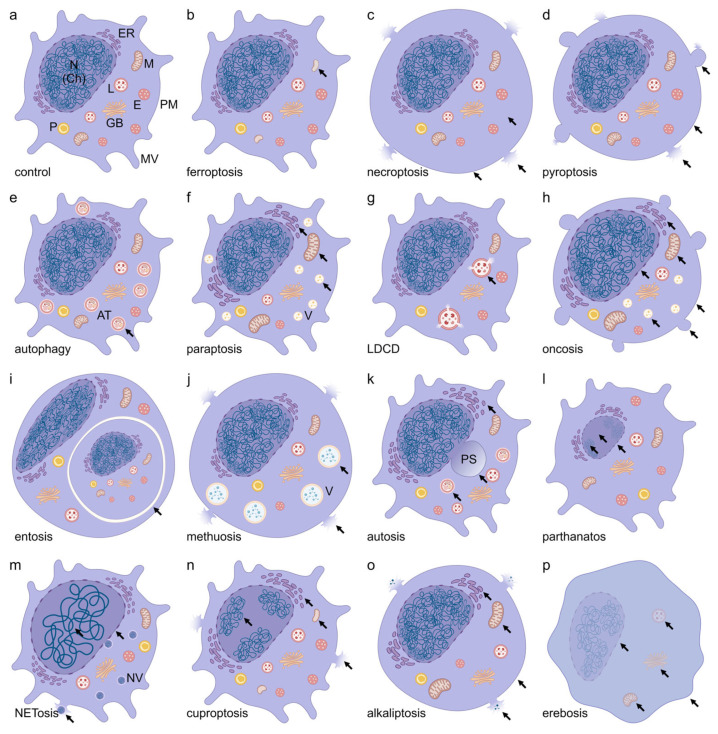
Schematic illustration of the known intracellular, ultrastructural hallmarks of the different representatives of non-apoptotic PCD. (**a**) Control cell: This cell represents a control cell with standard morphology and distribution of organelles and cell compartments such as the nucleus (N) including chromatin structure (Ch), endoplasmic reticulum (ER), mitochondria (M), lysosomes (L), endosomes (E), plasma membrane (PM), Golgi body (GB), peroxisomes (P), and microvilli (MV). (**b**) Ferroptosis: Decrease in mitochondrial volume and reduction in cristae structure. (**c**) Necroptosis: Cytoplasmic swelling, cell rounding, and ultimate rupture of the plasma membrane. (**d**) Pyroptosis: Cell swelling and plasma membrane ballooning with ultimate plasma membrane rupture. (**e**) Autophagy: Degradation of cytoplasmic components via increased number of autophagolysosomes (AT). (**f**) Paraptosis: Cytoplasmic vacuolation (V) and ER and mitochondrial swelling. (**g**) Lysosome-dependent cell death: Lysosomal swelling and lysosomal membrane permeabilization. (**h**) Oncosis: Mitochondrial-, ER- and cellular swelling, cytoplasmic vacuolization and blebbing, and nuclear dilatation. (**i**) Entosis: Invagination of viable cells into neighboring cells. (**j**) Methuosis: Accumulation of large fluid-filled macropinosome-like vacuoles and ultimate rupture of the plasma membrane. (**k**) Autosis: Ballooning of the perinuclear space (PS), ER dilatation and fragmentation, and autophagolysosome accumulation. (**l**) Parthanatos: Nucleus shrinkage, chromatin condensation, and DNA fragmentation. (**m**) NETosis: Swelling of the nucleus with chromatin decondensation, formation of NET-vesicles (NV), ER fragmentation, and ultimate plasma membrane rupture with NET-vesicle release. (**n**) Cuproptosis: Mitochondrial shrinkage with cristae degradation, plasma membrane rupture, ER swelling and fragmentation and unique alteration of chromatin structure. (**o**) Alkaliptosis: Mitochondrial and ER swelling, rounding of the cell and rupture of the plasma membrane with release of DAMPs. (**p**) Erebosis: Flattened nucleus (hard to observe), shortening of microvilli and reduction or loss of mitochondria, ER and Golgi-apparatus. Characteristic morphological alterations of the individual non-apoptotic PCD modalities are highlighted with arrows. Due to insufficient literature information on clear intracellular ultrastructural hallmarks, the following non-apoptotic PCDs were not included in the figure: Disulfidptosis, lysozincrosis, oxeiptosis, and pyronecrosis. Figure created with biorender.com.

Numerous approaches have been proposed in the literature for classifying non-apoptotic PCD. However, due to the rapid advancements in this field, such classifications often require revisions or updates. In this review, we describe and discuss the following currently predominant groups of non-apoptotic programmed cell death: ferroptosis, necroptosis, pyroptosis, autophagy, paraptosis, lysosome-dependent cell death, oncosis, entosis, methuosis, autosis, parthanatos, NETosis, metal-driven cell death, alkaliptosis, oxeiptosis, erebosis, and pyronecrosis. These non-apoptotic PCDs span a historical range from the first description of autophagy in 1859 by M. Anselmier [25] to novel characterizations such as “disulfidptosis” in 2023 [26]. Table 1 chronologically depicts the first historical description of each non-apoptotic PCD, based on publication year and corresponding literature reference(s). Table 2 provides an overview of the known and established pharmacological modulators for the different non-apoptotic PCDs as well as the relevant literature. Crucially, many pharmacological modulators may exhibit off-target effects due to the shared nature of signaling nodes (e.g., kinases) and metabolic processes (e.g., ROS generation, iron metabolism) across different cell death pathways. Therefore, to exclude potential cross-reactivity, pharmacological observations should ideally be validated using genetic interference approaches, such as CRISPR/Cas9 or RNAi silencing of the respective target proteins [1,27]. In addition to that, special focus is given on the intracellular known (ultra) structure based on organelle and compartment alterations and interactions corresponding to the non-apoptotic classifications. This is schematically depicted in Figure 1 and the known ultrastructural characteristics of the non-apoptotic PCD modalities are compared with a schematic control cell (Figure 1a). The selection of these non-apoptotic PCD modalities was based on established therapeutic relevance in overcoming apoptosis resistance in cancer and neurodegenerative diseases, availability of well characterized molecular mechanisms and pharmacological modulators that enable targeted interventions, and inclusion of both extensively studied modalities that have progressed to clinical trials, and recently characterized modalities that represent emerging therapeutic opportunities [1,2,13,26].

**Table 1:** Chronological first appearance of the different non-apoptotic programmed cell death variants based on the publication year and the corresponding publication(s) and ultrastructural references (regardless of the year). PCD modalities for which no or insufficiently characteristic ultrastructures could be assigned are listed as “not known” (n.k.).

Non-Apoptotic PCD	Year	Historical Reference	Ultrastructural Reference
Autophagy	1859	Anselmier 1859; de Duve 1963; Ktistakis [25]	[28]
Lysosome-dependent Cell Death	1965	de Duve [29]; Ondrouskova et al. [30]	[31]
Oncosis	1910/1995	Recklinghausen 1910 [32]; Majno et al. [33]	[34]
Paraptosis	2000	Sperandio et al. [35]	[36]
Pyroptosis	2001	Cookson et al. [37]	[38]
Necroptosis	2005	Degterev et al. [39]	[40]
Pyronecrosis	2007	Willingham et al. [41]	[42]
Entosis	2007	Overholtzer et al. [43]	[44]
NETosis	2007	Fuchs et al. [45]; Steinberg and Grinstein [46]	[47]
Parthanatos	2008	Andrabi et al. [48]	[49]
Methuosis	2008	Overmeyer et al. [50]	[51]
Ferroptosis	2012	Dixon et al. [5]	[52]
Autosis	2013	Liu et al. [53]	[54]
Oxeiptosis	2018	Holze et al. [55]	n.k.
Alkaliptosis	2018	Song et al. [56]	[56]
Lysozincrosis	2021	Du et al. [57]	n.k.
Cuproptosis	2022	Tsvetkov et al. [58]	[58]
Erebosis	2022	Ciesielski et al. [59]	[59]
Disulfidptosis	2023	Liu et al. [26]	n.k.

### 4.1. Ferroptosis

Ferroptosis is a distinct form of non-apoptotic PCD characterized by iron-dependent lipid peroxidation [60,61]. Intracellular hallmarks are mainly restricted to mitochondria, which decrease in volume and show a reduction in cristae structure [52,62] (Figure 1b). Since its discovery in 2012 [5] (Table 1), research on ferroptosis has grown exponentially, revealing its importance in various biological processes and diseases [63]. Key regulators of ferroptosis include RSL3, ML162, FIN56, erastin, sorafenib, sulfasalazine, deferoxamine, deferiprone, deferasirox, ferrostatin-1, liproxstatin-1, and vitamin E [5,60,63,64,65,66] (Table 2). Recent studies have identified new players in ferroptosis regulation, such as ferroptosis suppressor protein 1 (FSP1) and acyl-CoA synthetase long-chain family member 4 (ACSL4) [63,64]. Ferroptosis has been implicated in cancer, neurodegenerative diseases, ischemia–reperfusion injuries and inflammatory conditions [61]. The field has seen advancements in understanding the role of specific lipids, subcellular organelles, and metabolic pathways in ferroptosis. Importantly, ferroptosis induction has emerged as a promising strategy for treating therapy-resistant cancers, while its inhibition shows potential in treating degenerative diseases [64].

### 4.2. Necroptosis

Necroptosis is a programmed form of necrosis, or inflammatory cell death, distinct from apoptosis and unprogrammed necrosis. This results in a rounding of the cell due to cytoplasmic swelling, which can even lead to rupture of the plasma membrane [40,67] (Figure 1c). Necroptosis was first described in 2005 by Degterev et al. [39] (Table 1). It is mediated by receptor-interacting protein kinases (RIPK1 and RIPK3) and mixed lineage kinase domain-like protein (MLKL), which form a complex called the necrosome, and can be modulated by according inhibitors [68,69]. Modulators of necroptosis (necrostatin-1, ponatinib, pazopanib, GSK’872, GSK’843, TAK-632, and necrosulfonamide) are summarized in Table 2. This pathway is typically triggered by tumor necrosis factor (TNF) and other immune ligands, leading to cell membrane permeabilization and release of damage-associated molecular patterns (DAMPs) that elicit immune responses [70,71]. Recent studies have highlighted necroptosis as a defense mechanism against viral infections and its role in inflammatory diseases such as Crohn’s disease, pancreatitis, and myocardial infarction [72,73,74]. Necroptosis has also been implicated in neurodegenerative diseases like Alzheimer’s, where it is triggered by specific proteins in brain cells [75]. The potential for necroptosis-based therapies is being explored, particularly in cancer treatment, where inducing necroptosis in apoptosis-resistant tumors could enhance antitumor immunity [76].

### 4.3. Pyroptosis

Pyroptosis is a highly inflammatory form of non-apoptotic PCD characterized by cell swelling and plasma membrane ballooning, membrane rupture (Figure 1d), and the release of proinflammatory cytokines [38] and was first mentioned in 2001 [37,77,78] (Table 1). This process is primarily mediated by the activation of inflammatory caspases (caspase-1, -4, -5 in humans, and caspase-11 in mice) and the subsequent cleavage of gasdermin D, which forms pores in the cell membrane, leading to cell lysis and the release of IL-1β and IL-18 [79,80]. Pharmacologically relevant modulators of pyroptosis include MCC950, disulfiram, VX-765, Ac-YVAD-cmk, necrosulfonamide, and dimethyl fumarate [81,82,83,84] (Table 2). Recent studies have expanded our understanding of pyroptosis beyond its initial definition, revealing its role in various diseases, including cancer, cardiovascular disorders, and neurodegenerative conditions [80]. Notably, the high-resolution structure of the gasdermin D pore was solved using cryo-electron microscopy in 2021, providing new insights into the mechanism of pore formation [85]. Additionally, NINJ1 has been identified as a new molecule required for plasma membrane rupture during pyroptosis [86]. The potential of pyroptosis as a target for therapeutic interventions ranges from the treatment of non-infectious inflammatory diseases to strengthening antitumor immunity in cancer therapy.

### 4.4. Autophagy

Non-apoptotic autophagy is a critical cellular process that involves the degradation of cytoplasmic components via autophagolysosomes through the lysosomal machinery, making it distinct from apoptosis regulation (Figure 1e) [28]. In 1859, M. Anselmier used the term “autophagy” at the organism level [25]; later, in 1963, C. de Duve established this term at the cellular level (Table 1). Recent studies have highlighted the interplay between autophagy and other non-apoptotic cell death pathways, such as necroptosis, pyroptosis, and ferroptosis, revealing that autophagy can both promote and inhibit these forms of cell death depending on the context [87]. For instance, autophagy regulates necroptosis by degrading key necroptotic signaling molecules, thus preventing cell death. Additionally, autophagy can act as a scaffold for signaling complexes that modulate pyroptosis and ferroptosis, demonstrating its versatile role in cell death regulation. The most important pharmacological levers for manipulating autophagy are rapamycin, bafilomycin A1, 3-methyladenine, spautin-1, and metformin [88,89,90] (Table 2). The dual functions of autophagy in degradation and signaling underscore its complexity in maintaining cellular homeostasis and responding to stress [91]. Understanding these mechanisms offers potential therapeutic avenues for diseases where dysregulated cell death is a hallmark, such as cancer and neurodegenerative disorders [92].

### 4.5. Paraptosis

Paraptosis, a caspase-independent PCD mechanism, is characterized by cytoplasmic vacuolation and organelle swelling in the ER and mitochondria, lacking apoptotic hallmarks like nuclear fragmentation [36,93,94] (Figure 1f). These characteristics were described by Sperandio et al. in 2000 [35] (Table 1). Triggered by ER stress, Ca^2+^ overload, ROS, or IGF1R-MAPK/JNK signaling, this process requires de novo protein synthesis and involves regulators such as SHP2 and prohibitin [94,95]. Known pharmacological modulators of paraptosis are curcumin, celastrol, elaiophylin, paclitaxel, ophiobolin A, and cycloheximide [96,97] (Table 2). Morphologically, vacuole formation arises from ER/mitochondrial dilatation due to osmotic imbalance and Ca^2+^ flux. Therapeutically, paraptosis induction via nanomedicines, copper complexes, or shikonin shows promise in apoptosis-resistant cancers by exploiting iron metabolism dysregulation and EMT-related vulnerabilities [93,95,98]. Recent studies emphasize its role in overcoming chemoresistance, though biomarker development remains challenging due to overlapping pathways with autophagy and cuproptosis (see below). Precision targeting of paraptosis, particularly through combinatorial therapies, could enhance anticancer strategies while mitigating off-target effects [93,94,98].

**Table 2:** Established pharmacological modulators and their molecular targets (in brackets) of the individual non-apoptotic PCD sub-modalities and relevant literature references. No pharmacological modulators (and respective molecular targets) are currently known for erebosis.

Non-Apoptotic PCD	Pharmacological Modulators (Molecular Targets)	Reference
Ferroptosis	RSL3 (GPX4), ML162 (GPX4), FIN56 (GPX4), erastin (SLC7A11), sorafenib (SLC7A11), sulfasalazine (SLC7A11), deferoxamine (Fe^3+^), deferiprone (Fe^3+^), deferasirox (Fe^3+^), ferrostatin-1 (lipidperoxide), liproxstatin-1 (lipid radicals/lipidperoxide), vitamin E (lipid radicals/lipidperoxide).	[5,65,66]
Necroptosis	Necrostatin-1 (RIPK1), ponatinib (RIPK1/3), pazopanib (RIPK1), GSK’872 (RIPK3), GSK’843 (RIPK3), TAK-632 (RIPK1/3), necrosulfonamide (MLKL).	[68,69,99,100,101]
Pyroptosis	MCC950 (NLRP3), disulfiram (GSDMD), VX-765 (Caspase-1), Ac-YVAD-cmk (Caspase-1), necrosulfonamide (GSDMD), dimethyl fumarate (GSDMD)	[81,82,83,84]
Autophagy	Rapamycin (mTORC1), bafilomycin A1 (V-ATPase), 3-methyladenine (Vps34), spautin-1 (USP10/13), metformin (AMPK).	[88,89,90]
Paraptosis	Curcumin (Proteasom), celastrol (Proteasom), elaiophylin (MAPK), paclitaxel (microtubuli), ophiobolin A (KCNMA1), cycloheximide (ribosomes).	[96,97]
Lysosome-Dependent Cell Death	Siramesine (sigma-2receptor), chloroquine (lysosomes), bafilomycin A1 (V-ATPase), E64d (cathepsine B, H, L), CA074Me (cathepsine B), pepstatin A (cathepsine D, E), hydroxychloroquine (lysosomes), mefloquine (lysosomes), terfenadine (lysosomes).	[102,103,104,105,106]
Oncosis	GNE-617 (NAMPT), artesunate (ROS), aspirin (COX-1/2), dihydrotanshinone (Porimin), solamargine (lysosomes), sanguinarine (Na^+^/K^+^-ATPase), hyperosmotic solutions (aquaporins).	[107,108,109,110]
Entosis	Y-27632 (ROCK), H-1152 (ROCK), E-cadherin (adherens junctions), blebbistatin (Myosin II), latrunculin B (actin), cytochalasin B (actin).	[111,112,113]
Methuosis	MIPP/MOMIPP (Ras/Rac1), CX-4945 (CK2), CX-5011 (CK2), DZ-514 (ROS-MKK4-p38), JH530 (ROS-MKK4-p38), 5-iodoindole (macropinocytosis), vacquinol-1 (MAP2K4).	[114,115,116,117,118,119]
Autosis	Digoxin (Na^+^/K^+^-ATPase), ouabain (Na^+^/K^+^-ATPase), neriifolin (Na^+^/K^+^-ATPase), Tat-Beclin 1 (GLIPR2), thapsigargin (SERCA).	[53,54,120]
Parthanatos	Olaparib (PARP1/2), talazoparib (PARP1/2), oxaliplatin (PARP1), PJ- 34 (PARP1/2), 3-aminobenzamide (PARP), β-lapachone (NQO1), deoxy-podophyllotoxin (PARP1).	[121]
NETosis	Phor-bol-12-myristate-13-acetate (PKC), ionomycin (Ca^2+^), A23187 (Ca^2+^), nigericin (NLRP3), hypochlorous acid (ROS/MPO), SCN−, SeCN−, (4-amino) TEMPO (ROS).	[122,123]
Cuproptosis	Elesclomol (FDX1), disulfiram (NPL4/ALDH), NSC319726 (ROS).	[124,125]
Lysozincrosis	ML-SAs (TRPML1), ML-SIs (TRPML1).	[57,125]
Disulfidptosis	BAY-876 (GLUT1), PX-12 (Trx1), PMX464 (Trx1/R), selenocystine (TrxR), auranofin (TrxR), WZ26 (TrxR1), EGCG (TrxR), DHEA (G6PD).	[126]
Alkaliptosis	JTC-801 (ATP6V0D1), arenobufagin (Na^+^/K^+^-ATPase).	[127]
Oxeiptosis	Sanguinarin (KEAP1-PGAM5-AIFM1), mitoquinon (ROS), 4-octylitaconat (NRF2), dasatinib (Src), phenethylisothiocyanate (ROS), alantolactone (NRF2), alloimperatorin (KEAP1).	[127,128,129]
Pyronecrosis	NLRP3 (inflammasome), lurasidone (NLRP3), paliperidone (NLRP3), E64d (cathepsin B), miraziridine A (cathepsines), cystatin (cathepsines).	[130,131]

### 4.6. Lysosome-Dependent Cell Death

Lysosome-dependent cell death (LDCD) is a distinct non-apoptotic PCD pathway, characterized by lysosomal swelling and lysosomal membrane permeabilization (LMP) and subsequent release of lysosomal hydrolases, particularly cathepsins, into the cytoplasm [31,132,133] (Figure 1g). C. de Duve laid the foundation for LDCD in 1965 with the term “suicide bags” [29] (Table 1). LDCD is nowadays well established regarding PCD topics [30]. This process triggers cytosolic proteolysis and cellular decline, independent of caspase activation [134,135]. LDCD encompasses multiple morphological outcomes ranging from necrotic to apoptosis-like features, with the extent of LMP determining the specific death phenotype [136,137]. Recent studies have characterized lysoptosis as a conserved LDCD subtype, predominantly mediated by cathepsin L release following disruption of endogenous protease inhibitors such as SERPINB3 [133,138]. The best researched drugs for LDCD include siramesine, chloroquine, bafilomycin A1, E64d, CA074Me, pepstatin A, hydroxychloroquine, mefloquine, and terfenadine [102,103,104,105,106] (Table 2). The therapeutic potential of LDCD has gained prominent attention in cancer treatment, where malignant cells exhibit increased lysosomal membrane fragility compared to “normal” cells [136]. Targeting lysosomal integrity through lysosomotropic agents or cathepsin modulators offers promising strategies for overcoming apoptosis-resistant tumor populations [136,139]. Furthermore, LDCD plays pivotal roles in neurodegenerative diseases, where lysosomal dysfunction contributes to protein aggregation and neuronal loss [140,141].

### 4.7. Oncosis

Non-apoptotic oncosis is characterized by cellular swelling, mitochondrial and ER swelling and dysfunction, cytoplasmatic vacuolization, cytoplasmatic blebbing, nuclear dilatation and ATP depletion, in contrast with the cell shrinkage observed in apoptosis [34,107] (Figure 1h). The term “oncosis” was used as early as 1910 in a monograph by F. D. von Recklinghausen (Table 1). However, a more detailed definition and classification as a non-apoptotic PCD form was established years later [33]. This energy-dependent process is mediated by several key molecular mechanisms, including the opening of the mitochondrial permeability transition pore (MPTP), which leads to rapid loss of mitochondrial membrane potential and cessation of ATP synthesis. The porimin receptor, a transmembrane mucin family protein, serves as a critical mediator of oncotic cell death by facilitating pore formation in the cell membrane upon ligand binding, thereby increasing membrane permeability and initiating cellular damage [142]. Unlike apoptosis, oncosis is associated with significant ROS production and release of damage-associated molecular patterns (DAMPs) that trigger inflammatory responses [143]. Over the years, various pharmacological substances have been discovered, such as GNE-617, artesunate, aspirin, dihydrotanshinone, solamargine, sanguinarine, and hyperosmotic solutions, which have a direct or indirect effect on triggering oncosis in cells [107,108,109,110] (Table 2). Recent investigations have highlighted oncosis as a promising therapeutic target in cancer research, particularly for overcoming apoptosis resistance, with compounds such as dihydrotanshinone demonstrating the ability to induce porimin-dependent oncosis through ROS-mediated mitochondrial dysfunction in non-small-cell lung cancer [107,142].

### 4.8. Entosis

Entosis is characterized by the active invasion of viable cells into neighboring cells, resulting in distinct cell-in-cell structures [43,44,113,144] (Figure 1i). This was shown by Overholtzer et al. in 2007 [43] (Table 1). Unlike classical phagocytosis, entosis involves the active invasion of living cells into their neighbors through adherens junction-mediated interactions, leading to the death of invaginated cells via autophagy-dependent pathways [145,146]. The molecular entosis-machinery is built on the RhoA-ROCK signaling pathway, which regulates actomyosin contractility within internalizing cells [113,147]. Differential RhoA activity between neighboring cells creates the mechanical imbalance that is necessary for cell engulfment, with p190RhoGAP serving as a critical regulator by spatially restricting contractile forces at cell–cell junctions [148]. The process involves E-cadherin and P-cadherin-mediated cell adhesions that facilitate cell invasion, while subsequent entotic cell death occurs through LC3-associated autophagy and lysosomal degradation mechanisms rather than canonical apoptotic pathways [145,146,148]. This non-cell-autonomous death mechanism has been proposed as Type IV PCD, distinguishing it from apoptosis, necrosis, and autophagic cell death [149]. Pharmacological induction of entosis is well described and can be triggered via Y-27632, H-1152, E-cadherin, blebbistatin, latrunculin B, and cytochalasin B [111,112,113] (Table 2). Furthermore, entosis exhibits a dual role in cancer biology, functioning both as a tumor suppressor, due to elimination of detached cells and as a potential oncogenic processor, promoting aneuploidy and metabolic adaptation [150]. It was also shown that entosis can be induced by metabolic stress, particularly glucose starvation, enabling cancer cells to survive nutrient-limiting conditions through neighbor cell cannibalism [151]. Entosis therefore has great potential both as a biomarker and as a therapeutic agent in cancer. Current research is specifically investigating the modulation of entosis as a novel approach in cancer research [150,152,153].

### 4.9. Methuosis

Methuosis is characterized by the accumulation of large fluid-filled vacuoles derived from macropinosomes, ultimately resulting in plasma membrane rupture and subsequent cell death [50,51,154,155] (Figure 1j). Unlike apoptosis, methuosis proceeds without cellular shrinkage, nuclear fragmentation, or chromatin condensation, and is not prevented by caspase inhibitors, which was demonstrated in 2008 [50,154] (Table 1). The process involves dysfunctional endocytic trafficking where macropinosomes fail to recycle to the plasma membrane or fuse with lysosomes, instead accumulating and coalescing into progressively larger vacuoles that displace cytoplasm [155,156]. Two distinct classes of methuosis have been identified as follows: Class I induced by oncogenic Ras activation through the Ras/Rac1/Arf6 signaling pathway, and Class II triggered by external compounds targeting endosomal trafficking components [127,156]. Recent advances in small-molecule methuosis inducers, including chalcone derivatives like MIPP and MOMIPP, have demonstrated selective cytotoxicity against cancer cells while sparing normal tissues, suggesting therapeutic potential for apoptosis-resistant malignancies [116,154,157,158]. Studies have shown promising preclinical efficacy in various cancer models, including glioblastoma, breast cancer, and hepatocellular carcinoma, with compounds like DZ-514 and vacquinol-1 demonstrating tumor suppression through methuosis induction [116,117,159]. The full spectrum of pharmacological manipulation of methuosis consists of MIPP/MOMIPP, CX-4945, CX-5011, DZ-514, JH530, 5-iodoindole, and vacquinol-1 (Table 2). The therapeutic appeal of methuosis lies in its ability to circumvent apoptotic resistance mechanisms commonly observed in cancer cells, offering an alternative cell death pathway for cancer treatment [127,157,160].

### 4.10. Autosis

Autosis is a distinct autophagy-dependent modality of non-apoptotic PCD that has played an important role in cell biological and biomedical research since its initial definition in 2013 [53] (Table 1). Its unique character is distinguished by characteristic morphological features, including ballooning of the perinuclear space, ER dilation and fragmentation, autophagolysosome accumulation, and enhanced cellular substrate adhesion [53,54,161] (Figure 1k). Unlike conventional apoptosis or necrosis, autosis is specifically regulated by the Na^+^/K^+^-ATPase pump and can be pharmacologically inhibited by cardiac glycosides without affecting other cell death pathways [53,162]. Mechanistically, the interaction between the Na^+^/K^+^-ATPase and Beclin 1 is pivotal, as its disruption by cardiac glycosides or specific genetic knockdown prevents the formation of autotic vacuoles and reduces tissue injury in cerebral and renal ischemia–reperfusion models [163]. Only recently it was shown that pharmacological treatment with the SERCA (Sarco/Endoplasmic Reticulum ATPase) inhibitor Thapsigargin can lead to autosis in immune cells [54]. Autophagy-inducing peptides have also been described as inducers of autosis along digoxin, ouabain, neriifolin, Tat-Beclin 1, and thapsigargin [53,120] (Table 2). Other recent investigations have identified tissue-specific molecular mechanisms, particularly the dependency on the ATP1A3 subunit in neuronal autosis rather than the ubiquitous ATP1A1 subunit found in other cell types [164]. The process involves excessive accumulation of autophagosomes mediated by dysregulated autophagic flux, often through upregulation of Rubicon during pathological conditions such as ischemia–reperfusion injury [162,165]. Furthermore, autosis has been documented in various clinical coherence including cardiac ischemia–reperfusion injury, neonatal hypoxic–ischemic encephalopathy, and renal injury, with evidence of ATP1A3-BECN1 interactions observed in post-mortem human brain tissue from newborns with severe hypoxic–ischemic encephalopathy [164]. These findings establish autosis as a therapeutically relevant target, with ongoing research exploring its selective activation in cancer cells and its potential inhibition in neurodegenerative and cardiovascular diseases [166,167].

### 4.11. Parthanatos

Parthanatos describes a caspase-independent PCD, mainly caused by DNA damage through nucleus shrinkage, chromatin condensation and DNA fragmentation [49,121] (Figure 1l). It is mediated by the overreaction of poly(ADP-ribose) polymerase 1 (PARP1), the resulting accumulation and release of PAR from the nucleus into the cytoplasm and the subsequent release of apoptosis-inducing factor (AIF) from mitochondria, which was first described in 2008 [48] (Table 1). Together with the macrophage migration inhibitory factor (MIF), AIF forms a complex whose translocation into the nucleus leads to the condensation of chromatin and fragmentation of DNA and thus to cell death [121,168,169]. Parthanatos seems to be energy dependent as a decrease in NAD^+^ and depletion of ATP can be observed [121] and is also accompanied by the loss of membrane potential [168]. Morphologically, this type of cell death shows similar features as necrosis or apoptosis, like loss of the integrity of the plasma membrane [121]. The role of parthanatos and its mediators PARP1, AIF, or ROS has been described in connection with various diseases such as cancer, heart diseases, retinal diseases, diabetes, and neurological diseases such as Alzheimer’s disease, Parkinson’s disease, or stroke, and therefore represents an interesting field of study. Since several PARP1 inhibitors (olaparib, talazoparib) are already approved by the Food and Drug Administration (FDA) for the treatment of ovarian or breast cancer (PJ-34 and 3-aminobenzamide are not FDA approved), and PARP1 agonists (β-lapachone, deoxypodophyllotoxin, oxaliplatin (secondary)) also show the potential of inducing parthanatos (Table 2), a better understanding of this PCD represents a potential for new therapeutic approaches [121].

### 4.12. NETosis

The non-apoptotic PCD modality NETosis, characterized in 2007 [45,46] (Table 1), is an inflammatory, NADPH-oxidase dependent form of cell death in which reticular structures consisting of granular proteins and chromatin histones, so-called neutrophil traps (NET), are released into the extracellular space by neutrophils [168,170,171]. Intracellularly, there is swelling of the nucleus with chromatin decondensation, formation of NET vesicles, ER fragmentation and a final plasma membrane rupture in which the NETs are released [47,122,172,173,174] (Figure 1m). Additionally, other immune cells like eosinophils, basophils, macrophages, or mast cells were observed releasing extracellular traps/extracellular DNA for which the terms EETosis (for eosinophils) or ETosis (in general) have been proposed [173,175]. Inducers of NETosis include bacterial toxins like ionomycin, ROS [173], pathogens like bacteria, viruses, or fungi [170]. In addition, the participation of several plasma membrane associated receptors (e.g., Toll-like receptor 2 (TLR2), interleukin-1 (IL-1) receptor or Fc receptors) is under discussion. During NETosis, cells tend to spread before shedding microvesicles (annexin V-positive) of their plasma membrane and finally rounding up [173]. At the ultrastructural level, Caruso et al. [176] observed roundish or oval nuclear lobes, increased perinuclear space, decondensation and expansion of chromatin, loss of plasma membrane integrity, and a higher number of free extracellular granules (FEGs) in early EETosis. In the intermediate stage, complete degranulation occurs, as well as the appearance of cytosolic decondensed chromatin (either as irregular blocks or serpeginous deposits), which are still connected by chromatin bridges. In the advanced stage, extracellular DNA traps (identified as chromatin aggregates connected with FEGs) could be observed, due to rupture of the plasma membrane. It should be noted that this was observed in 1 out of 4 gastric cancer patients and further research is needed to obtain conclusive results, as well as to determine the applicability of these findings in reference to NETosis or ETosis [176]. Only previously, a decrease in NET was shown after the use of antioxidants (e.g., SCN−, SeCN−), suggesting a direct effect on NETosis [123]. Other established modulators of NETosis (Phor-bol-12-myristate-13-acetate, ionomycin, A23187, nigericin, hypochlorous acid, (4-amino) TEMPO) are listed in Table 2. Nevertheless, further studies are required to identify possible use in therapeutic approaches.

### 4.13. Metal-Driven Programmed Cell Death: Cuproptosis, Lysozincrosis, and Disulfidptosis

Cuproptosis is considered a metal-driven PCD as excess cytosolic copper concentration leads to binding of copper to lipolyated mitochondrial enzymes (e.g dihydrolipoamind S—Acetyltransferase (DLAT)) associated with the tricarboxylic acid cycle (TCA). The subsequent accumulation of copper-bound proteins, in combination with the resulting reduction of Fe-S clusters, leads to proteotoxic stress, production of ROS and, consequently, to cell death [124,177,178,179]. Morphological hallmarks include mitochondrial shrinkage with cristae degradation, plasma membrane rupture, ER swelling and fragmentation, and a change in chromatin structure, which is distinct from apoptotic chromatin condensation [174,177,180,181] (Figure 1n). This was only recently first characterized in 2022 by Tsvetkov et al. [58] (Table 1). Interestingly, cell death triggered by elesclomol, a Cu-ionophore that increases intracellular copper, could not be prevented with inhibitors of ferroptosis, necroptosis, or apoptosis. Other potential modulators of cuproptosis are disulfiram and NSC319726 (Table 2). As an important micronutrient, copper is needed for a great variety of physiological processes, including its role as a co-factor for metabolic enzymes [124]. Furthermore, evidence shows that an increase in serum copper ions is associated with a variety of cancer types, for example lung cancer, breast cancer or thyroid cancer [179]. Additionally, other diseases like Wilson’s disease, Menkes disease, or neurodegenerative diseases show a dysregulation of copper [124,177]. Lysozincrosis and disulfidptosis are two newly described metal-induced PCD subtypes that are considered non-apoptotic [26,57,125,182] (Table 1). The mechanism behind lysonzincrosis involves an increase in cytosolic Zn^2+^ through lysosomal Zn^2+^ release, leading to the following toxic effects on other cell organelles like mitochondria. The lethal mechanism involves the rapid uptake of released Zn^2+^ into mitochondria, likely via the mitochondrial calcium uniporter (MCU), which induces severe mitochondrial metabolic dysfunction and ROS production [57]. Here, focus is on the cation channel TRPML1, which is mainly associated with the lysosomal membrane and is permeable to Ca^2+^ and Zn^2+^. Cells (e.g., metastatic melanoma cells [57]) undergoing lysonzincrosis show energy depletion and dysfunction in mitochondria, which in the long term results in cell death [57,125]. Potent modulators and therefore interesting compounds in cancer therapy are agonists or inhibitors of TRPML channels (ML-SAs, ML-SIs) [57,125] (Table 2). Disulfidptosis is triggered by the accumulation of disulfide molecules, which is accompanied by the depletion of NADPH and consequently leads to cell death [125,182]. Specifically, the excessive intracellular accumulation of disulfides causes aberrant disulfide bonding within the actin cytoskeleton (e.g., in ACTB and MYH9 proteins), triggering a rapid cytoskeletal collapse that is distinct from other cell death modalities [26]. Disulfidptosis is also described in connection with cancer cells that show a high concentration of SLC7A11 with simultaneous low-glucose conditions, while cells under the same low-glucose conditions but with a low SLC7A11 concentration undergo apoptosis. This makes this type of cell death particularly interesting in the context of cancer therapy [182]. Disulfidptosis is considered as a form of PCD caused by metabolic factors, in which excessive accumulation of cystine ultimately leads to cell death. Several ways of modulating disulfidptosis, targeting different key regulators, are described (BAY-876, PX-12, PMX464, selenocystine, auranofin, WZ26, EGCG, DHEA; Table 2). For example, the inhibition of the glucose transporter GLUT via GLUT-inhibitors or the inhibition of the Thioredoxin (Trx) system, seems to be a promising strategy in cancer therapy. Another strategy might be the inhibition of the NADPH metabolism and its regulators [126] (Table 2). Since disulfidptosis and lysozincrosis are relatively novel characterizations within non-apoptotic PCD, clear intracellular hallmarks have not yet been fully described. For this reason, these modalities are not included in Figure 1. However, this also highlights the importance of future cell biological research on this topic.

### 4.14. Alkaliptosis

Alkaliptosis is a pH-dependent form of regulated cell death that was first discovered and described in 2018 [56] (Table 1). Not only does it have distinct molecular markers compared to other forms of PCD, but its mechanism is also independent of genes that trigger apoptosis, necrosis, or autophagy [127]. Furthermore, known inhibitors of other cell death forms (e.g., “ferrostatin-1” (ferroptosis), “hydroxychloroquine (HCQ)” (autophagy) or “necrosulfonamide (NSA)” (necroptosis)) were not able to inhibit cell death induced by JTC-801 [56,127]. Alkaliptosis shares certain hallmarks with primary necrosis, e.g., morphological features such as mitochondrial and ER swelling, rupture of plasma membrane associated with the release of endogenous DAMPs, and cellular “rounding” [56,127] (Figure 1o). This form of cell death is considered proinflammatory, energy-dependent, and irreversible [127]. Two pathways are described in which cancer cells can undergo alkaliptosis: the NF-κB-CA9 pathway and the ATP6VOD1-STAT3 pathway. A defining feature of this modality is the profound intracellular alkalization (pH > 8.0) which disrupts metabolic enzyme function and activates the NF-κB-CA9 axis; this mechanism remains refractory to inhibitors of apoptosis, ferroptosis, or necrosis [56]. Both pathways can be triggered by the small-molecule compound JTC-801 (Table 2), which is an antagonist of OPRL1 [56,127,183]. Interestingly, JTC-801 shows no toxicity in normal “healthy” cells like hepatocytes, peripheral blood mononuclear cells, dermal fibroblasts, or bone marrow CD34^+^ progenitor cells compared to cancer cells such as pancreatic cancer cells, melanoma cells, kidney carcinoma cells, prostate cancer cells, and central nervous system cancer cells. In addition, the anticancer drug “arenobufagin” (ArBu) (Table 2) in combination with chemotherapeutics could be shown to be a very effective treatment against gastric cancer by triggering cell death in form of alkaliptosis [127]. Additional known modulators are, for this reason, the subject of numerous current studies that discuss the importance of alkaliptosis in connection with potential cancer therapies [56,127,183,184].

### 4.15. Oxeiptosis

ROS are associated with several cell death types like apoptosis, ferroptosis, or necroptosis. However, in 2018 a new ROS-induced, noninflammatory, caspase-independent cell death was described: Oxeiptosis [55] (Table 1). It is distinguishable from other cell death types as it states a distinct signaling pathway even though it shows an apoptosis-like morphology [55,127,129,185]. Since oxeiptosis is not clearly distinguished ultrastructurally from apoptosis, this non-apoptotic PCD was excluded from Figure 1. The pathway behind oxeiptosis is known as the KEAP1-PGAM5-AIFM1 signaling pathway. Interestingly, the “Kelch-like ECH-associated protein 1” (KEAP1), together with the transcription factor “nuclear factor erythroid-derived 2-like” (NRF2; also known as NFE2L2) show cytoprotective functions in oxidative injuries [129,185]. However, in oxeiptosis, induced by ROS, KEAP1 acts independently of NRF2, together with “phosphorylate mutase 5” (PGAM5) and “apoptosis-inducing factor mitochondria-associated 1” (AIFM1) [129,185]. Functionally, this pathway serves as a critical host defense mechanism against viral infections (e.g., Influenza A), where it restricts viral replication and limits excessive inflammation through the ROS-sensing capability of KEAP1 [55]. As ROS are involved in various diseases (e.g., Alzheimer’s disease or Parkinson’s disease), a better understanding of oxeiptosis could potentially be an interesting target in novel therapeutic approaches, since several compounds (sanguinarin, mitoquinon, 4-octylitaconat, da-satinib, phenethylisothiocyanate, alantolactone, alloimperatorin) that are currently under discussion have an influence on oxeiptosis [127] (Table 2).

### 4.16. Erebosis

Erebosis (greek: “erebos”, which means “complete darkness”), was first described in 2022 by Ciesielski et al., whose team discovered this process of cell death in gut enterocytes of Drosophila [59,170] (Table 1). This modality of PCD is ultrastructurally characterized by a flattened nucleus that can be hard to detect, as well as the reduction or loss of mitochondria, ER and Golgi-apparatus, and shortening of microvilli [59] (Figure 1p). It is theorized that erebosis might be important for the gut barrier function and for maintaining a continuous flux of the tissue [59]. The principle that a layer of dead cells represents an important mechanical protective barrier of the skin has already been described in context with cornification in which terminal differentiated keranocytes represent the uppermost skin layer of the epidermis (*stratum corneum*) [186,187]. The features of cells undergoing erebosis do not appear to be comparable to those of apoptosis, autophagy, or necrosis. The cells show a flat morphology, loss of proteins and organelles [170], reduced nuclear lamin with a larger nuclear diameter (compared to surrounding enterocytes), weak or an absence of fluorescence response upon DAPI or Hoechst staining, which could be due to loss of DNA or closed chromatin structure [59], and the accumulation of angiotensin-converting enzyme (ACE or Ance in Drosophila) [59,170]. This process is presumably driven by a distinct metabolic state characterized by the specific accumulation of Ance and depletion of cytoskeletal components, facilitating the homeostatic turnover of enterocytes without disrupting the integrity of the gut barrier [59]. As research into this type of cell death is still in its early stages, further studies are needed to better understand the relationship between erebosis, its function, its pharmacological manipulation, and its role as a potential target for novel therapeutic approaches, especially in gut diseases [170].

### 4.17. Pyronecrosis

Pyronecrosis is a necrosis-like, proinflammatory, lytic cell death type dependent on an inflammasome complex [168,171,188]. Several pathogens such as *Shigella flexneri* and *Neisseria gonorrhoeae* have been identified as triggers of pyronecrosis. Furthermore, *Toxoplasma gondii parasitophorous*, *Staphylococcus aureus,* and *Bacillus anthracis* lethal toxin are also suspected of causing cell death, which could be referred to as pyronecrosis [171]. This was first observed and defined in 2007 by Willingham et al. [41] (Table 1). This process is mediated by formation of the inflammasome complex by the oligomerization of the “NLR family pyrin domain containing 3” receptor (NLRP3), which in turn leads to the oligomerization of “apoptosis-associated speck-like protein containing a caspase-recruitment domain” (ASC) [168]. Other studies also name the lysosomal protein cathepsin B, which is also an important factor in pyronecrosis [171,188]. The formation of the complex induces not only the production and release of proinflammatory cytokine interleukin-1β (IL-1β), but also the release of the proinflammatory factor “high-mobility group box 1” (HMGB1) [168,171]. This process is suspected to end with the swelling of the cell, followed by rupture of the cell membrane, and the subsequent release of intracellular content. Although this has been shown in an ultrastructural context [42], current studies suggest that the clear (ultrastructural) features (especially in contrast to LDCD and pyroptosis) are missing [1,31,189], and so a schematic representation of pyronecrosis in Figure 1 was omitted. Cryopyrin-associated periodic syndrome (CAPS), a rare autoinflammatory disease caused by a gain-of-function mutation in the protein NLRP3, is mentioned in connection with the activation and formation of the NLRP3-dependent inflammasome [190]. Therefore, a better understanding of pyronecrosis could be beneficial for a better understanding and therapeutic approaches of inflammatory diseases like CAPS. Furthermore, possible pharmacological regulators of proteins are important for pyronecrosis (NLRP3, lurasidon, paliperidon, E64d, miraziri-dine A, cystatin; Table 2), and are a potential research topic. For example, lurasidone and paliperidone, both with antipsychotic and antineurotic abilities, show inhibitory effects on CTSB [130]. However, despite their theoretically great potential, their roles in pyronecrosis have not yet been fully investigated.

## 5. Discussion

In modern biomedical research, non-apoptotic PCD has become important for understanding cell regulation and therapeutic intervention [2]. The recognition of new non-apoptotic PCD subtypes by the NCCD in 2018 was a literal starting point for the study of such mechanisms beyond classical apoptosis [1]. But even apart from and especially after this recognition, there have been extensive innovations and changes surrounding existing or newly characterized non-apoptotic PCD modalities [26,59,76]. Recent advances demonstrate that non-apoptotic PCD pathways offer compelling therapeutic opportunities in overcoming apoptosis resistance [191,192]. Combining nanomedicine and non-apoptotic PCD inducers has revolutionized targeted drug delivery, enabling selective tumor elimination with less off-target effects [2,15,16,17,193]. Clinical translation is advancing rapidly, with multiple ongoing trials evaluating non-apoptotic PCDs across various cancer types. The emerging concept of PANoptosis has further illuminated pathway interconnectivity [191,194]. As crosstalk mechanisms deepen understanding, combinatorial strategies targeting multiple death modalities simultaneously show enhanced efficacy in preclinical models. However, successful clinical implementation requires pathway-specific biomarker development and precision medicine approaches based on individual tumor vulnerabilities. Regardless of the detailed characterization of these modalities, critical knowledge gaps remain regarding their hierarchy and crosstalk in complex tissue environments. Future research must prioritize the development of highly specific, validated biomarkers and the use of combinatorial genetic models (e.g., multiplex CRISPR screening) to dissect pathway interconnectivity and functional redundancy in vivo [195]. Despite the promising preclinical data, the clinical translation of non-apoptotic cell death modulators faces significant hurdles, primarily due to the lack of validated biomarkers for patient stratification and the poor pharmacokinetic properties of many current experimental compounds. Furthermore, the complexity of the tumor microenvironment and potential compensatory mechanisms between different cell death pathways require the development of more sophisticated, likely combinatorial or correlative, therapeutic strategies and advanced drug delivery systems to ensure specificity and efficacy [1,185,196]. The field of non-apoptotic PCD is undoubtedly a rapidly evolving and active area of biomedical research. To keep pace with this change, updated overviews are of great value. In addition to this, “templates” based on cellular or intracellular hallmarks of the corresponding non-apoptotic PCDs can also assist in their initial interpretation and might help answer a wide variety of open biomedical questions.

## Data Availability

No new data were created or analyzed in this study.

## Abbreviations

The following abbreviations are used in this manuscript:

ACE	Angiotensin-converting enzyme
ACSL4	Acyl-CoA Synthetase long-chain Family Member 4
ACTB	Actin Beta
Ac-YVAD-cmk	Acetyl-Tyr-Val-Ala-Asp-chloromethyl ketone
AIF	Apoptosis-inducing factor
AIFM1	Apoptosis-inducing factor mitochondria-associated 1
ALDH	Aldehyde Dehydrogenase
AMPK	AMP-activated protein kinase
ASC	Apoptosis-associated speck-like protein containing a caspase-recruitment domain
ATP	Adenosin Triphoshpate
ATP1A1	ATPase Na+/K+ transporting subunit alpha 1
ATP1A3	ATPase Na+/K+ transporting subunit alpha 3
ATP6V0D1	V-type proton ATPase subunit d 1
BECN1	Beclin 1
CAPS	Cryopyrin-associated periodic syndrome
CK2	Casein Kinase 2
COX-1/2	Cyclooxygenase-1/Cyclooxygenase-1
CRISPR	Clustered Regularly Interspaced Short Palindromic Repeats
CTSB	Cathepsin B
DAMPs	Damage-associated molecular patterns
DHEA	Dehydroepiandrosterone
DLAT	Dihydrolipoamind S—Acetyltransferase
DNA	Deoxyribonucleic acid
EET	Eosinophil traps
EGCG	Epigallocatechin Gallate
EMT	Epithelial–mesenchymal Transition
ER	Endoplasmic Reticulum
FEGs	Free extracellular granules
FDA	Food and Drug Administration
FDX1	Ferredoxin 1
FSP1	Ferroptosis Suppressor Protein 1
GLIPR2	GLI Pathogenesis-Related 2
GLUT	Glucose Transporter type
GPX4	Glutathione Peroxidase 4
GSDMD	Gasdermin D
G6PD	Glucose-6-Phosphate Dehydrogenase
HCQ	Hydroxychloroquine
HMGB1	High-mobility group box 1
IGF1R	Insulin-like Growth Factor 1
IL	Interleukin
JNK	c-Jun N-terminal kinase
KCNMA1	Potassium Calcium-Activated Channel Subfamily M Alpha 1
KEAP1	Kelch-like ECH-associated protein 1
LC3	Microtubule-associated protein 1 light chain 3
LDCD	Lysosome-dependent cell death
LMP	Lysosomal membrane permeabilization
MAPK	Mitogen-Activated Protein Kinase
MCU	Mitochondrial calcium uniporter
MIF	Migration inhibitory factor
MIPP	3-(2-methyl-1H indol-3-yl)-1-(4-pyridinyl)-2-propen-1-one
MKK4	Mitogen-Activated Protein Kinase 4
MLKL	Mixed Lineage Kinase domain-like
MOMIPP	3-(5-methoxy, 2-methyl-1H-indol-3-yl)-1-(4-pyridinyl)-2-propen-1-one
MPO	Myeloperoxidase
MPTP	Mitochondrial permeability transition pore
m-TORC1	Mammalian Target of Rapamycin Complex 1
MYH9	Myosin Heavy Chain 9
NAD^+^	Nicotinamide Adenine Dinucleotide
NADPH	Nicotinamide Adenine Dinucleotide Phosphate
NAMPT	Nicotinamide Phosphoribosyltransferase
NCCD	Nomenclature Committee on Cell Death
NET	Neutrophil traps
NF-κB-CA9	Nuclear Factor kappa B—Carbonic Anhydrase 9
NINJ1	Ninjurin-1
NLR	nucleotide-binding oligomerization domain, leucine-rich repeat
NLRP3	NLR family pyrin domain containing 3
NPL4	Nuclear protein localization protein 4 homolog
NQO1	NAD(P)H:quinone oxidoreductase 1
NRF2	Nuclear factor erythroid-derived 2-like
NSA	Necrosulfonamide
PARP	Poly(ADP-ribose) polymerase
PCD	Programmed Cell Death
PGAM5	Phosphorylate mutase 5
PKC	Protein Kinase C
RIPK	Receptor-interacting Protein Kinase
RNAi	Ribonucleic Acid interference
ROCK	Rho-associated protein kinase
ROS	Reactive Oxygen Species
RSL3	(1S,3R)-RAS-selective lethal compound 3
SERCA	Sarco/Endoplasmic Reticulum ATPase
SHP2	Src homology 2 domain-containing protein tyrosine phosphatase 2
SLC7A11	Solute Carrier Family 7 Member 11
TCA	Tricarboxylic acid cycle
TEMPO	(2,2,6,6-Tetramethylpiperidin-1-yl)oxyl
TLR2	Toll-like receptor 2
TNF	Tumor Necrosis Factor
TRPML1	Transient Receptor Potential Mucolipin 1
TrxR	Thioredoxin Reductase
Trx-1	Thioredoxin-1
USP10/13	Ubiquitin-specific peptidase 10/Ubiquitin-specific peptidase 13
